# A Study of Theory of Mind in Paranoid Schizophrenia: A Theory or Many Theories?

**DOI:** 10.3389/fpsyg.2012.00432

**Published:** 2012-11-14

**Authors:** Peter Scherzer, Edith Leveillé, André Achim, Emilie Boisseau, Emmanuel Stip

**Affiliations:** ^1^Département de Psychologie, Université du Québec à MontréalMontréal, QC, Canada; ^2^Institut des Sciences Cognitives, Université du Québec à MontréalMontréal, QC, Canada; ^3^Hôpital Rivière-des-PrairiesMontréal, QC, Canada; ^4^Centre de Recherche Fernand Séguin de l’Hôpital Louis H. LafontaineMontréal, QC, Canada

**Keywords:** schizophrenia, paranoid symptoms, theory of mind, overmentalization, undermentalization, test specificity

## Abstract

Social cognitive psychologists (Frith, 1992; Hardy-Baylé et al., 2003) sought to explain the social problems and clarify the clinical picture of schizophrenia by proposing a model that relates many of the symptoms to a problem of metarepresentation, i.e., theory of mind (ToM). Given the differences in clinical samples and results between studies, and considering the wide range of what is considered to constitute ToM, one must ask if there a core function, or is ToM multifaceted with dissociable facets? If, there are dissociable dimensions or facets, which are affected in patients with paranoid schizophrenia? To answer these questions, a group of 21 individuals diagnosed with paranoid schizophrenia and 29 non-clinical control subjects, were tested on a battery of five different measures of ToM. The results confirmed that there was little difference in specificity of three of the tests in distinguishing between the clinical and non-clinical group, but there were important differences in the shared variance between the tests. Further analyses hint at two dimensions although a single factor with the same variance and the same contributing weights in both groups could explain the results. The deficits related to the attribution of cognitive and affective states to others inferred from available verbal and non-verbal information. Further analyses revealed that incorrect attributions of mental states including the attribution of threatening intentions to others, non-interpretative responses and incomplete answers, depending on the test of ToM.

## Introduction

### Theory of mind

Theory of mind (ToM) is that cognitive function that allows an individual to attribute information, beliefs, intentions, or feelings to others, in order to explain and eventually predict their behavior. This construct is both clinically and theoretically significant as it furnishes a basis for the explanation of autistic semiology (Baron-Cohen et al., 1985) as well as other clinical populations such as Asperger’s syndrome (Happé et al., 1996) and dementia (Gregory et al., 2002). Not only does this construct describe a complex behavior but it also furnished new substance for the study of normal development (see Wimmer and Perner, 1983; Frith and Frith, 2003) and the neuroanatomic basis of this development (e.g., Saxe and Powell, 2006). As such, the concept is an essential tool that allows researchers and clinicians to better understand normality, autism spectrum as well as other pathologies in which social dysfunction is an important symptom, notably schizophrenia.

### Schizophrenia and theory of mind

Frith (1992) postulated that that deficits in meta representation are at the origin of all cognitive aspects of schizophrenic symptomatology (p. 122) which include mentalization or ToM. Schizophrenic patients with negative symptoms are noted for poverty of content, unawareness of intentions (p. 114), flattened affect, and social withdrawal (Bodlakova et al., 1974). Others have described a lack of ToM (Shamay-Tsoory et al., 2007) errors of literal interpretations of mental states or overly simplistic inferences (Frith, 2004; Montag et al., 2011, 2012). This characterization is supported by data showing that schizophrenic patients tend to describe the physical appearance of peoples’ photographs rather than their state of mind (Pilowsky and Bassett, 1980; Allen, 1984). They make overly simplistic interpretations of state of mind of others, or ignore completely the other’s state of mind (Montag et al., 2011). These symptoms are also related to flattened affect, social withdrawal and chronicity (Bodlakova et al., 1974). In contrast with these symptoms, Frith (2004) described another class of errors such as errors in the prediction of behavior based on wrong beliefs, or ascribing significance to what others perceive as incidental or random events (see also Blakemore et al., 2003) Other descriptions include increased self-reference, an excessive interpretation, or over attribution of beliefs or knowledge or a mental state to another (Frith, 2004), overgeneralization of hypotheses, or a hyper-ToM (Frith, 1994; Abu-Akel and Bailey, 2000; Abu-Akel, 2003; Montag et al., 2012) or social inferential reasoning that goes beyond the bounds of the context (Montag et al., 2011). Other manifestations include increased self-reference (Frith, 2004), delusional beliefs, delusions of reference, hallucinations, persecutory thoughts and disorders monitoring, and interpreting others thoughts and intentions (Frith, 1992; Abu-Akel and Bailey, 2000).

The Frith model of mentalization deficits in schizophrenia is partially supported by the Bora et al. (2009a,b) and Sprong et al. (2007) meta-analyses. In the latter case, the clinical groups were found to be more than one SD below the mean of the control subjects on different measures of ToM (*d* = −1.13). The ToM deficit was more marked in the disorganized type of schizophrenia (*d* = −2.23) and significantly greater than that of schizophrenic subjects presenting a negative (*d* = −1.28) or paranoid symptomatology (*d* = −1.24) or in remission (*d* = −0.692). Other studies showed negative correlations between the severity of delusions and performance on the Hinting Task (Greig et al., 2004), between the severity of symptoms of persecution and the results on a test of first and second degree false beliefs (Harrington et al., 2005) and between the severity of paranoid symptoms and the results on a test of sarcasm (Kern et al., 2008). Finally, the performance of paranoid schizophrenic (PScz) subjects on the Hinting Task is significantly impaired compared with schizophrenic patients with negative symptoms (Bora et al., 2008) and with schizophrenic patients in remission on the Hinting Task, and tests of inferences of mental states derived from cartoon strips (Corcoran et al., 1995, 1997). On the other hand Kosmidis et al. (2011) found group differences in a first order false belief cartoon task, a hinting task, first and second order false belief-verbal and deception-verbal tasks but not on a task of attribution of intention or desire.

In their review of the literature, Couture et al. (2006) proposed a model of social cognition and social functioning in schizophrenia similar to Frith (2004) in which a deficit of social cognition is at the origin of social disturbance in schizophrenic patients. This model is partially supported by correlations between measures of social cognition such as emotional and social perception, ToM as well as styles of attribution, and measures of social functioning in a hospital or community setting, social competence, and social problem solving (Penn et al., 1997; Keltner and Kring, 1998; Couture et al., 2006; Meyer and Kurtz, 2009). Paranoid schizophrenia patients share these characteristics but present particular social and cultural characteristics that distinguish them from the general population of patients with schizophrenia and other clinical populations. Studies have found a better ability to perceive facial expressions of fear, anger, and sadness in patients with PScz, compared with non-PScz patients (Penn et al., 1997, 2008; Keltner and Kring, 1998; Green and Phillips, 2004). As well, patients with delusions of persecution require more time to name the color of a threatening word compared with a neutral word or a word related to depression and they tend to have a better recall of threatening words and propositions. They also tend to find significant associations between random words compared with non-PScz patients (Penn et al., 1997; Green and Phillips, 2004).

### Methodological issues

To date, although studies of ToM and PScz patients seem to arrive at the same findings of deficits, methodological differences would tend to limit their construct validity. Deficits in ToM have been related to positive (Mehl et al., 2010a) or negative (Kelemen et al., 2005) or positive and negative (Greig et al., 2004) symptoms of schizophrenia, to paranoid delusions (Mehl et al., 2010a), or not (Greig et al., 2004), to the absence of a relationship between schizophrenia and ToM (Brüne, 2005; Montag et al., 2011). These apparent contradictions can be attributed to differences in the questions asked, in clinical samples (size, age, diagnosis – Kettle et al., 2008) and differences in test batteries with the incumbent distinctions in the processes that are involved in the specific ToM task (Apperly, 2008).

From a methodological perspective, there is the problem of the measure of ToM when the format is texts. This format solicits cognitive functions other than ToM, such as reading ability, autobiographical memory, and working memory, the latter two being impaired in schizophrenic patients (Daum, 2008; Mehl et al., 2010b). Other cognitive functions that are related to reading texts in general and have been found specifically in ToM are IQ and executive functions (Corcoran et al., 1995). However, there is some dispute over whether or not they adequately explain performance on ToM tests (Corcoran et al., 1995; Penn et al., 1997; Langdon and Coltheart, 1999; Brüne, 2003; Greig et al., 2004; Brüne and Bodenstein, 2005; Pickup, 2006; Brüne et al., 2008). See however Pickup and Frith (2001) for contrary findings.

Despite the link between ToM abilities in schizophrenia and social abilities (Bora et al., 2008; Mehl et al., 2010b; Montag et al., 2011), several teams of researchers have elaborated measures of ToM that have been found to be more ecologically valid with different clinical groups (Colussy and Zaruff, 1985; Davis and Stewart, 2001; Dziobek et al., 2006; Bazin et al., 2009; Bell et al., 2010; Mehl et al., 2010a; Ouellet et al., 2010). These tasks consist of structured interviews (e.g., Bosco et al., 2008; Morgan and David, 2010) or more generally of a video presentation of real people in interaction in social situations. As in any real-life situation, the information to be processed is complex, in real time, involving visual and verbal information with the interpretation at times being determined by the context. One would expect such complexity to increase their sensitivity to detect an impairment in social cognition in a clinical population (Colussy and Zaruff, 1985; Davis and Stewart, 2001; Bell et al., 2010).

### Hypotheses

It is hypothesized that the performance of the clinical group will be significantly impaired compared to that of the non-clinical group on all five ToM tests. It is also postulated that the clinical group will produce more incorrect attributions, excessive interpretation, or over attribution of knowledge or a mental state to the third party, than unawareness of intentions, errors of literal interpretations of mental states, incomplete, or overly simplistic inferences, inasmuch as an attempt was made to limit the negative symptoms in the clinical group, relative to positive symptoms. We also postulate that an ecological measure, Conversations and Insinuations (C&I), a real-life test of ToM appropriate for an adult population, will be more sensitive to deficits of ToM than questionnaires or texts, in the clinical group. Equally important, given the wide variety of ToM tests used, of the diverse cognitive functions solicited by each test, we hypothesize that the correlations between ToM tests will be weak or not significant, thus raising the question of the existence of different modules of ToM, some of which are more impaired PScz than others.

## Materials and Methods

The Comité institutionnel de déontologie et de la recherche de l’Hôpital Louis-H-L a Fontaine and the institutionally delegated departmental Comité de déontologie of the Université du Québec à Montréal approved of this project and all subjects gave their free and informed written consent to participate in the study. The treating psychiatrist of each patient took the necessary steps to ensure that the patients were able to understand their rights, and what was entailed and that their consent was freely given.

### Participants

Two groups of participants, PSczs and a non-clinical group were recruited for the study. The group of 21 patients was recruited from the outpatient clinic Jeunes adultes of the Hôpital Louis-H-L a Fontaine by their respective psychiatrists. The clinical participants had to have been diagnosed with active (not in remission) PScz by a psychiatrist, according to the American Psychiatric Association (1994) DMS-IV diagnostic criteria. They had to obtain a score of >4 on the scale of positive and negative symptoms (PANSS) and on one or more of the following symptoms: delusions, ideas of grandeur, or of persecution/suspicion. There had to be no change in their antipsychotic medication for at least 2 weeks before their participation and during their participation in this study.

The 29 non-clinical control subjects were recruited from the community via posters and in discussions with groups of individual. They had to have a sociodemographic profile (age, education, family education) comparable to that of the clinical group and no member of their direct family (parents, brothers, sisters) could have been diagnosed with schizophrenia at any time prior to their recruitment. They were recruited into the study following a telephone interview of ∼20 min during which time they were questioned about their personal and medical history in order to ensure that they did not report any signs of Axis 1 or Axis 2 pathology, head trauma, or meet other exclusionary criteria.

All subjects were males, aged between 18 and 35, with French their first language or language of instruction. Their IQ (VIQ, PIQ, FSIQ) had to be ≥85. As well there could not be any Axis I or II comorbidity, any neurologic impairment, or dysfunction, uncorrected visual defects, alcoholism, or addiction and they could not be under the influence of recreational drugs or alcohol during testing, as confirmed by a psychiatrist.

### Material

#### Evaluation of the symptomatology

The severity of the symptomatology of the clinical subjects was determined using the Positive and Negative Syndrome Scale (PANSS; Kay et al., 1986) during a semi-structured interview. This scale is composed of three subscales measuring positive, negative, and general psychopathology. The 30 items of the Scale are scored on a 7-point Likert scale ranging from absence of an invalidating symptom in the past week to presence of such a symptom. Each PANSS score was verified by an independent psychiatrist.

#### IQ

The participants’ IQ was determined using the abridged French version of the Wechsler Adult Intelligence Scale (WAIS-III; Wechsler, 1997). VIQ, PIQ, and FSIQ were determined according to the following formula using raw scores (Pilgrim et al., 1999):

$$\begin{array}{rcll} & \text{VIQ}=2\left(\text{Information}+\text{Similarities}\right)+\left(\text{Digit Span}+\text{Arithmetic}\right) & & \\ & \text{PIQ}=\text{2}\left(\text{Picture completion + Block design}\right)+\left(\text{Substitution}\right) & & \end{array}$$

FSIQ is calculated according to the traditional method using the scaled scores of the two indices.

#### Evaluation of theory of mind

##### Reading the mind in the eyes test

This test is sensitive to subtle ToM deficits in a population of adults with autism spectrum disorder (Baron-Cohen et al., 2001). According to the authors it measures the ability to infer the mental state without having to infer the content (Baron-Cohen et al., 2001; Bora et al., 2008). The test consists of 36 images of pairs of men’s and women’s eyes, presented one at a time to the subject who then has to choose between four adjectives describing the cognitive or affective mental state of the person behind the eyes. In this study we used a French version of the test (Audrey Simion, www.autismresearchcenter.com). One point is given for each correct answer for a total of 36 points.

##### Hinting task

This test has two versions of 10 stories each, describing social interactions in which one person sends an indirect message to another (e.g., a business man makes a subtle comment to a colleague meant to tell him to let him rest for a few minutes after having spent a long and hot day on the road; Corcoran et al., 1995; Marjoram et al., 2005). Language is used to convey a message that is not explicitly expressed, requiring certain knowledge of the context to be able to correctly infer the intended message. The stories are read to the subject who can follow along on his copy of the text. The subject has to identify the indirect message when first asked. If the answer is wrong, a cue is give and the subject is asked a second question. Two points are given for the unaided answer for a total of 40 points, one point for the correct answer when cued, zero points if the answer is still wrong after the cue.

The first version of this test was developed by Corcoran et al. (1995) to investigate ToM in schizophrenic patients and the second version was developed by Marjoram et al. (2005). In the latter study the second version was significantly more difficult than the first for both the clinical group and a non-clinical control group. In this study a French translation of the two versions was used. They were first translated by Emilie Boisseau then verified by Peter Scherzer and whatever discrepancies remained were resolved following a discussion between the two. Each wrong answer to the first question is independently scored by two judges on the basis of three types of errors. There are non-interpretative errors, errors of incomplete or implicit attributions, and errors of incorrect attributions (Snowden et al., 2003).

##### Strange stories

Strange Stories has been used to investigate ToM in a variety of populations. The test consists of eight stories requiring social inferences (e.g., a captured soldier tells his captors where his army’s assault vehicles are located knowing that his captors will think that he is lying in order to protect his army) and eight stories requiring physical inferences (Happé and Wimmer, 1998). The stories are read to the subjects who can follow along on their copy of the text. The subject has to explain the behavior of the protagonist and answer a question. In this study a French version, validated using the translation-retranslation method, was used. Two points are given for a correct answer for a wrong answer for a total of 16 points, one point for an incomplete or ambiguous answer and zero points.

##### Faux pas

This test is composed of 10 stories describing social interactions in which one person, unintentionally says something that is hurtful to another person (e.g., the winner of a writing contest tells another contestant that it was easy to win because the other stories were terrible, not knowing that this person was also a contestant; Stone et al., 1998). The stories are read to the subjects who can follow along on their copy of the text. After hearing and reading each story, the subject is asked eight questions. A score of one is given for each correct answer to each of six questions for each faux pas for a maximum of 60 points and a score of zero is given in the case of a wrong answer. The participant who receives one point for question 4 (a woman threatens to drown a kitten if nobody wants to buy it) automatically receives one point for question 5. Subjects can then receive 20 more points for their written comprehension of questions 7 and 8. Finally, each wrong answer to question 4 is first categorized by two judges according to whether it is incorrect, incomplete, or literal (Snowden et al., 2003). The errors are then categorized as referring to internal states, personality traits, bad, i.e., wrong intentions, the wrong good intentions, or answers “don’t know” (Zalla et al., 2008).

This test is sensitive to subtle ToM deficits. The understanding and correct interpretation of faux pas requires the ability to attribute a cognitive and affective mental state to the person making the faux pas and the recipient of the comment (Stone et al., 1998). The English version was translated by Emilie Boisseau then verified by Peter Scherzer. The few discrepancies were resolved following a discussion between the two.

##### Conversations and insinuations

This test is a more real-life test of ToM appropriate for an adult population (Ouellet et al., 2010). The four clips that make up the test came from popular daytime TV shows. Each clip is approximately 2 min in length, with two to four characters (wife and husband, brother and sister, grandmother and grandson, roommates) interacting in a variety of real-life situations (restaurant, hospital room, apartment, kitchen). The subject has to make inferences in order to correctly understand the social interactions as the TV characters make use of indirect messages, faux pas, irony, and lies. Each scene is independent of the rest of that episode with no references to preceding episodes or other characters (see Ouellet et al., 2010 for a more complete description).

There are three to six preprogrammed pauses for each video clip with the pauses timed to occur after a social behavior or interaction, in order to ask the subject to explain what the subject did, said, or feels. When questioned, the subject first answers spontaneously and then is given a multiple-choice question, regardless of the first answer. The test is made up of a total of 21 multiple-choice questions. These questions always ask for an interpretation of the implicit message: of something said [“Why do you think that he (she) said that”?], of an action [“why do you think he (she) did that”?], or of a feeling [“How do you think that he (she) feels now?”], depending on the content of the clip. Among the choice of responses there is the correct response and three wrong answers, presented in random order. The categories of wrong answers are derived from Rowe et al. (2001). There are three types of wrong answers: (1) non-interpretative (2) attribution of an erroneous mental state (3) an inappropriate response. The wrong response choices to questions related to the recognition of an affective mental stage refer to a plausible but less precise emotion.

Two points are accorded to a correct response, one point for an incomplete or inaccurate response that nevertheless has elements of the correct response and zero if the answer is wrong. The subject receives a supplementary point if he chooses the correct response to the multiple-choice question. A total score is calculated for each video clip for a grand total of 63 points. The sensitivity of this test has already been demonstrated in a study of patients with multiple sclerosis and a frontal lobe semiology (Ouellet et al., 2010).

#### Procedure

The test battery was given over two sessions of ∼2 h each. The sequence of ToM tests was counterbalanced between subjects. The clinical participants were tested at the Center de recherche Fernand–Séguin of the Hôpital Louis-H-Lafontaine and the control subjects were tested at the Université du Québec à Montréal. All the participants received a remuneration of $8/h to compensate them for their time, travel, and any inconvenience (Note: the ethics committee of the Hôpital Louis H-Lafontaine required that all subjects be treated equally).

## Results

### Data distributions

Data analysis was preceded by inspection of within group distributions. Reading the Mind in the Eyes Test (RMET), Hinting Task and C&I were reasonably symmetrical, with their skewness index being smaller that one standard error in each group. The skewness index exceeded two standard errors in the control group for Faux Pas (2.96, *z* = −6.82) and for Strange Stories (−0.931, *z* = −2.15) while that for the patient group was acceptable (0.795, *z* = −1.59, and 0.396, *z* = 0.79, respectively). The transformations 2-log10(60.5-Faux Pas) and 5-sqrt(22-Strange Stories) preserved the polarity of the scores and gave acceptable skewness in each group, namely, for Faux Pas, 0.135 (*z* = 0.27) and −0.468 (*z* = 1.08) in the patient and control groups respectively, and for Strange Stories, 0.621 (*z* = 1.24) and −0.579 (*z* = −1.33) for patients and controls.

### Subject characteristics

Student *t*-tests revealed significant differences between the clinical and non-clinical groups. The clinical group was older, less educated, and their IQ was lower (Table 1). However, there is no evidence that an age and education difference of 2 years might affect the results. Global IQ and schooling are significantly correlated with all ToM tests with the exception of RMET (Table 2) and were inserted as covariables in all analyses. A partial correlation analysis between ToM scores in the clinical group and demographic data and PANSS scores revealed significant correlations after controlling for FSIQ and schooling. There was a significant correlation between RMET and symptoms of hostility (*r* = 0.73, *p* = 0.00), as well as between the performance on C&I and symptoms of hallucinations (*r* = −0.63, *p* = 0.02).

**Table 1:** **Group demographic data and clinical characteristics of schizophrenic patients**.

	Clinical	Non-clinical	*t*(48)
	*n* = 21	*n* = 29	
	*M* (SD)	*M* (SD)	
Age	25.71 (4.44)	23.07 (3.2)	2.45*
Education (subjects)	10.71 (1.55)	12.03 (1.18)	−3.42**
Education (parents)	11.94 (2.06)	12.91 (2.45)	−1.38
FSIQ	99.95 (11.58)	110 (11.04)	−3.11**
VIQ	100.81 (11.84)	110.14 (13.90)	−2.47*
PIQ	98.24 (12.09)	111.03 (13.83)	−3.40**
Age at diagnosis	21.57 (2.50)		
Chronicity (year)	5.67 (5.13)		
PANSS positive symptoms (0–49)	21.19 (2.46)		
-delusions (0–7)	4 (1.34)		
-thoughts of grandeur (0–7)	2.38 (0.74)		
-ideas of persecution/ suspicion (0–7)	3.86 (1.15)		
PANSS negative symptoms (0–49)	17.48 (4.72)		
PANSS psychopathology (0–112)	39.43 (6.67)		

*Between-group comparisons: two-tailed t-tests for independent samples*.

*p < 0.05, **p < 0.01.

**Table 2:** **Correlation matrix of ToM test scores and subject characteristics**.

	RMET	Hinting task	Strange stories	Faux pas	C&I
Age	0.08	−0.26	−0.02	0.20	−0.19
Schooling	0.11	0.39**	0.30*	0.35*	0.33*
FSIQ	0.18	0.53**	0.52	0.31*	0.58**

*p < 0.05, **p < 0.01.

**Table 3:** **Pooled within groups correlation matrix**.

	Test	RMET	HT	C&I	ST	FP
Correlation	RMET	1	−077	0.111	−0.033	−0.098
	HT	−0.077	1	0.464	0.445	0.330
	C&I	0.111	0.464	1	0.558	0.311
	ST	−0.033	0.445	0.558	1	0.405
	FP	−0.098	0.330	0.311	0.405	1

*RMET, Reading the Mind in the Eyes Test; HT, Hinting Task; C&I, Conversations and Insinuations; ST, Strange Stories; FP, Faux pas*.

*RMET is not significant, the other correlations are significant at *p* < 0.05*.

### Analyses of ToM measures

The extent to which the five tests of ToM together distinguish between groups was examined using a MANOVA using transformed scores where applicable. This discriminated very well between the groups (Wilks λ = 0.006, *F*(5,44) = 1470.1, *p* < 0.0005. The individual variables gave the following results: hinting task, *F*(1,48) = 87.66, *p* < 1e−8; C&I, *F*(1,48) = 57.13, *p* < 1e−8; Faux pas, *F*(1,48) = 51.45, *p* < 1e−8; Strange Stories, *F*(1,48) = 15.28, *p* < 3e−7; RMET *F*(1,48) = 1.177, *p* = 0.283. RMET was the only measure that did not distinguish between the two groups.

### Individual analyses of ToM tests

#### Reading the mind in the eyes test

There was no significant difference in the performance on this test between the two groups.

#### Faux pas

The performance of the clinical group was significantly impaired compared with the non-clinical group on the Faux Pas test. Tests of comparison of the differences in proportions revealed that the clinical group had more difficulty than the non-clinical group recognizing social faux pas (Q1: *z* = −5.87, *p* < 0.01), indicating who made the faux pas and explain what it was (Q2-3: *z* = −11.82, *p* < 0.01) and explain what the aggrieved party felt (Q6: *z* = −2.83, *p* < 0.01).

The percentage of correct answers was particularly low (22%) when the clinical group was asked to explain why the individual faux pas (Q4) compared with the non-clinical group (83%). When the participants gave an incorrect answer they were asked a supplementary question in order to clarify their understanding of the faux pas (Q5). The percentage of correct answers then increased to 63.4% in the clinical group and 95.4% in the non-clinical group.

#### Strange stories

The performance of the clinical group was significantly impaired compared with the non-clinical group on Strange Stories. An ANCOVA, controlling for FSIQ and schooling was used to compare the performance on GROUPS (Clinical, Non-Clinical) and STORIES (mental states, physical inferences) with repeated measures on the latter. The analysis revealed a main effect of GROUP only [*F*(1,46) = 23.63, *p* = 0.00, *d* = 1.73] with no interaction effect. The performance of the clinical group was impaired [Mental States: *M* = 10.66 (SD = 3.51); Physical inferences: *M* = 9.29 (SD = 2.89)] compared with the non-clinical group [Mental States: *M* = 14.90 (SD = 1.38); Physical inferences: *M* = 11.79 (SD = 2.63)]. A test of comparison of the differences in proportions of the number of correct and wrong answers was then carried out. The clinical group made significantly less correct inferences of mental state and physical interferences (56.0 vs. 45.2%) than did the non-clinical group (92.7 vs. 71.1%; Mental States: *z* = −8.73, *p* < 0.01; Physical inferences: *z* = −5.32, *p* < 0.001).

#### Conversations and insinuations

The performance of the clinical group was significantly impaired compared with the non-clinical group on C&I. An ANCOVA, controlling for FSIQ and schooling was used to compare the performance on GROUPS (Clinical, Non-Clinical) and ANSWER (spontaneous, multiple-choice) with repeated measures on the latter. The analysis revealed a significant main effect for GROUP [*F*(1,46) = 40.47, *p* = 0.00], and ANSWER [*F*(1,46) = 5.29, *p* = 0.03] but no interaction effect. The clinical group was significantly impaired compared with the non-clinical group regardless of the type of answer and both groups improved significantly when presented with multiple-choice questions.

#### Hinting task

The performance of the clinical group was significantly impaired compared with the non-clinical group on the Hinting Task. An ANCOVA, controlling for FSIQ and schooling was used to compare the performance on GROUPS (Clinical, Non-Clinical) and VERSIONS (1, 2), with repeated measures on the latter. There was a significant main effect for GROUP only [*F*(1,46) = 61.63, *p* = 0.00, *d* = 1.82]. The performance of the clinical group was significantly impaired on both versions [Version 1: *M* = 15.39(SD = 2.54); Version 2: *M* = 14.00(SD = 1.01)] compared with the non-clinical group [Version 1: *M* = 18.48(SD = 2.33); Version 2: *M* = 18.20(SD = 1.40)]. Tests of comparison of the differences in proportions of the number of correct and wrong answers were then carried out. The clinical group made significantly less correct inferences in social situations than did the non-clinical group (*z* = −11.32, *p* < 0.01). Although the percentage of correct inferences increased in both groups when clues were given, the clinical group still made less correct social inferences, 89 vs. 98.8%.

### Qualitative analyses

#### Hinting Task

Two judges made an analysis of the three types of errors prior to the cues and the results are considered acceptable (κ = 78.62%). Further tests of comparisons (contingencies and uniform adjustments) did not reveal any difference between the groups (χ^2^ = 3.63, *p* = 0.163) for any of the three types of errors, all subjects confounded (χ^2^ = 3.92, *p* = 0.141). The non-clinical group made the same percentage of non-interpretative errors (19 vs. 30%), incomplete attributions (47 vs. 35%), and incorrect attributions (33 vs. 35%) as the clinical group.

#### Strange stories

Two judges made an analysis of the percentage of the three types of errors for the social inference questions and the results are considered acceptable (κ = 84.85%). Tests of comparisons (contingencies and uniform adjustments) did not reveal a pattern difference between groups [χ^2^(2) = 0.19, *p* = 0.91] but did show a significant difference between the three types of errors, when the data from both groups is combined [χ^2^(2) = 9.54 *p* = 0.009]. There were significantly more non-interpretative (Σ = 36), and incorrect attributions (Σ = 28), than errors of incomplete attributions (Σ = 14). As well a supplementary analysis of the fourth story of mental states attribution revealed that of the 21 clinical participants, 14 were unable to correctly explain this story and of these, nine thought that the woman was in fact going to drown the kittens. Only two of the 29 non-clinical participants were unable to correctly explain this story but none thought that the woman would really drown the kittens. The story was chosen for a more detailed analysis as the content allowed us to explore in more details if the participants in the clinical group were more prone to an interpretation that one could interpreted as paranoid ideation, the participants’ belief that the woman really had a bad intention toward the kittens (the intention of the woman to kill).

#### Faux pas

Two judges made an analysis of the percentage of the three types of wrong answers to the fourth question and the results are considered acceptable (κ = 81.19%). Tests of comparisons of percentages (contingencies and uniform adjustments) did not reveal any significant difference between the two groups [χ^2^(2) = 3.57, *p* = 0.17] but did reveal significant differences between the three types of errors, all subjects confounded [χ^2^(2) = 46.31, *p* < 9e−11]. The percentage of non-interpretative errors (Σ = 65), was significantly greater than the percentage of errors of incorrect attributions (Σ = 37), that was significantly greater than the percentage of errors of incomplete attributions (Σ = 7).

The two judges made a second analysis of the five types of errors made on the fourth question and the results for errors of incomplete attributions and incorrect attributions are considered acceptable (κ = 98.42%). Tests of comparisons of percentages (contingencies and uniform adjustments) revealed significant differences between the two groups. The clinical group had a significantly greater percentage of errors of “Bad intentions” (35 vs. 10, *z* = 2.04, *p* = 0.04), “Don’t know” (17 vs. 0, *z* = 3.04, *p* = 0.0024), and “Good intentions,” (8 vs. 0, *z* = 1.98, *p* = 0.049) while there was no difference between the two groups for errors “Internal states” (20 vs. 40, *z* = −1.15, *p* = 0.25) and “Personality traits” (20 vs. 50, *z* = −1.70, *p* = 0.09).

#### Conversations and insinuations

Two judges made an analysis of the percentage of the three types of wrong answers and the results are considered adequate (κ = 81.61%). Tests of comparisons of percentages (contingencies and uniform adjustment) revealed a pattern difference between the two groups [χ^2^(2) = 10.61, *p* = 0.005]. The percentage of non-interpretative errors made by the clinical group was significantly proportionally greater than that of the non-clinical group [χ^2^(1) = 7.37, *p* = 0.007]. As well, the clinical group made significantly proportionally more errors of incorrect attributions, than incomplete attributions [χ^2^(1) = 15.11, *p* = 0.0001]. The percentage of errors of incomplete attributions, however, was strictly proportional to group size [χ^2^(1) = 0.006, *p* = 0.94].

### Analyses of ToM construct

Pooled within group correlations (i.e., correlations between the variables without the effect of group), were obtained from a discriminant analysis for the purpose of later decomposition by principal component analysis. The following correlation matrix was obtained, where the critical values of the correlation coefficient at the 0.05 and 0.01 levels are respectively 0.2816 and 0.3646 for a two-tailed test.

The discriminant analysis replicated the MANOVA but further identified the dimension beneath the group difference. While the structure matrix (correlations of the canonical variable with the observed variables) had only positive values (Hinting Task: 0.832; C&I: 0.672; Faux Pas: 0.637; Strange Stories: 0.347; RMET: 0.096), the standardized canonical discriminant function coefficients (i.e., the weights to apply to the standardized observed variable to estimate the subjects’ scores on the canonical variable) included one negative weight (Hinting Task: 0.663; C&I: 0.406; Faux Pas: 0.447; Strange Stories: −0.351; RMET: 0.135). A divergence in sign between the correlation and the coefficient for the same observed variable is indicative of a suppressor variable effect. This effect is, in this context, the presence of a source of information that does not contribute to discriminating the groups but contaminates other variables in expressing the underlying dimension on which the groups differ, making them less predictive than they would otherwise be. Note that from the structure matrix, that Hinting Task, with its correlation of 0.83 is the variable closest to the underlying dimension.

To pursue this interpretation, step-wise logistic regression was applied. At step 0, the table of variables not in the equation indicated that RMET does not discriminate the groups (*p* = 0.274) while the remaining four variables do so at *p* < 0.001. In the forward step-wise mode, after the Hinting Task entered, all remaining measured had *p* > 0.10. Thus, no other measure significantly reduced the log-likelihood index, meaning that this variable captures the essence of what distinguishes the patients from the controls. But because a suppressor effect was suspected, the backward step-wise mode was called for. Reading Mind in Eyes was first eliminated, followed by C&I, non-significantly increasing the chi squared fit index (−2 log-likelihood) by 0.60 and 2.27 respectively, for 1 degree of freedom each. At this point, removing either Strange Stories or Faux Pas, in the presence of the other, increased the chi squared by 4.72 (*p* = 0.03) or 5.40 (*p* = 0.02). Applied to scores standardized over all 50 participants (i.e., irrespective of group membership, the predictive equation is:

$$\begin{array}{rcll} 8.33*z\left(\text{Hinting Task}\right) & -2.42*z\left(\text{Strange Stories}\right) & & \\ & +2.81*z\left(\text{Faux Pas}\right). & & \end{array}$$

Thus Strange Stories and Faux Pas appear to each contain two sources of information, where an appropriate combination of them cancels the dimension not discriminating the groups but leaves information that reinforces group discrimination. The latter source of information does not have to be distinct from that represented in the Hinting Task measure; it could be that it reduces the estimation error contained in Hinting Task.

While analyses centered on functions that best differentiate the groups indicate two underlying dimensions with a suppressor variable effect, analyses based on the pooled within group correlation matrix do not confirm this feature. As seen above, the pooled correlation matrix indicate that RMET does not correlate significantly with any other variable while all inter-correlations among the remaining four variables are significant and positive.

Principal component analysis on all five measures hint at two dimensions (by the Scree test and by producing two eigenvalues above 1.0), but one dimension consists essentially of RMET. When this variable is taken out, the first two eigenvalues are 2.27 and 0.73, and the Scree test supports that there is only one dimension.

The number of dimensions was further questioned with structural equation modeling of the two covariance matrices (excluding the RMET variable), despite clearly too small sample sizes. This indicated that a single factor with the same variance and the same contributing weights in both groups is an acceptable model (χ^2^ = 12.8, df = 8, *p* = 0.12), although the RMSEA of 0.158 indicates lack of power to detect eventual model inadequacies.

## Discussion

### Profile of quantitative and qualitative deficits on ToM tests

In this study, several steps were taken to control for sources of variance that might affect internal validity: these included homogeneity of diagnosis, the relatively young age of patients, limited chronicity, normal IQ, stability of medication prior to, and during testing, mainly positive symptomatology during testing. It should be noted that there were differences in cognitive functioning between the two groups that might explain some of the differences obtained on the ToM tests. These differences included VIQ, PIQ, FSIQ and differences between the groups on the non-ToM tests. After controlling for the confounding IQ factors, the performance of the PScz patients remained impaired on four of the five measures of ToM, relative to the non-clinical group. While this basic result confirms those found in many previous studies (Corcoran et al., 1995; Randall et al., 2003; Harrington et al., 2005; Langdon et al., 2006; Bora et al., 2008; Montag et al., 2011), it does not reveal much about the structure of ToM nor its clinical significance in paranoid schizophrenia.

Of the five ToM tests, only the RMET did not distinguish between the clinical and non-clinical groups. This result is in agreement with Bora et al., 2008, but conflicts with that of other studies (see for example Kelemen et al., 2005; Hirao et al., 2008; Kettle et al., 2008; Bora et al., 2009a,b) which did find significant impairment in PScz patients with this test. The explanation for this seemingly paradoxical result is most likely found in the performance of the non-clinical group. For reasons unknown, the performance of this group of subjects [*M* = 21.68 (SD = 3.61)] is poorer than that found in previous studies: de Achával et al., 2010, *M* = 27.3 (3.8); Bailey and Henry, 2010, *M* = 27.3 (3.40); Craig et al., 2004, *M* = 27.63 (4.33); Riveros et al., 2009, *M* = 27 (4.16); Schimansky et al., 2010, *M* = 25.5 (2.6), although there is only one SD between this result and those reported by Schimanskty et al. and at most 1.65 SDs between this study and the others cited (see Craig et al.).

From the point of view of what the results reveal about the structure of ToM, what should first be the difference in the amount of shared variance between the tests (*r*^2^). Faux Pas shared the least variance with the other tests (excluding RMET), with a shared variance ranging from 9.67% (Faux Pas and C&I: *r* = 0.311,) to 20.25% (Faux Pas and Strange Stories: *r* = 0.405). In comparison, the shared variance between Strange Stories and the other measures of ToM (excluding RMET) ranged from 10.80% (Strange Stories and Hinting Task: *r* = 0.445) to 31.15% (Strange Stories and C&I: *r* = 0.558). The shared comparison between Strange Stories and C&I contrasts with that between Faux Pas and C&I and what is also notable is the difference in the pattern of correlations between tests. One evident explanation for these results is that the tests measure different dimensions of ToM in the Hinting Task it is indirect messages, Faux Pas is self-explanatory. in Strange Stories it is white lies, double bluffs, jokes, irony, figures of speech, and misunderstandings; in the C&I it is faux pas; indirect messages, lies, jokes, figures of speech, irony. Despite the fact that the mode of presentation of Strange Stories and C&I differ, the two tests assess a large variety of ToM dimensions and share similarities, sufficient to explain why they share 31% of the variance (*r* = 0.558). The absence of most of these dimensions in the other tests and the unique social cognitive ToM dimension targeted in two (faux pas, indirect messages), would explain the weakness of the correlation between these tests and the others. Although the tests purport to measure a common concept, ToM, some tests measure a unique social cognitive ToM dimension and these dimensions appear tobe dissociated. The pattern of correlations found in this study is partially supported by the results in the Kosmidis et al. (2011) study. These authors used seven measures of ToM in 28 patients with schizophrenia and 30 non-clinical controls. Not all measures distinguished between the groups and not all ToM measures were related in the clinical group. They concluded that not all aspects of ToM were impaired, and the deficits were more isolated and specific in their group, i.e., a dissociation in ToM dimensions.

The postulate that ToM is multidimensional is not novel. Shamay-Tsoory et al., 2007) and Montag et al., 2011 identified a cognitive and an emotional dimensions. Bell et al. (2010) classify ToM (and the tests used) into a mixture of content and context. In their classification system of content, they identify a social cognitive dimension and a social perceptual dimension.

Duval et al. (2011) present a different perspective, a model of ToM that is composed of three modules, the first being composed of two sub-modules first order of cognitive second order of cognitive representations of the other. Next is a module of representations of the intentions of the other then a module that is the affective representations of the other. Faux pas would fit into the module of first and second order representations, understanding of intentions (“why did X say that?”) and emotions (“How does Y feel when s/he hears what X said?”). Next, Faux Pas shares the least variance with the other measures, confirming its independence from the other modules, One might conclude that patients with paranoid schizophrenia have some difficulties identifying socially embarrassing situations in others (and most likely identifying it in themselves; sub-module of affective representations of the other) but this difficulty in social cognition is not as marked as their other social cognitive difficulties. Their greatest difficulty is interpreting contradictory messages, by messages that are not explicitly, specifically expressed, that have to be contextualized in order to be able to correctly infer the intended message.

The hypothesis of dissociable ToM dimensions was further tested in this study, using a discriminant analysis, principal component analysis and structural equation modeling. The results furnish only indirect support for the hypothesis of multiple ToM dimensions. Principal component analysis on all five measures hint at two dimensions although further questioning the number of dimensions with structural equation modeling, despite the small sample sizes, indicate that a single factor would be an acceptable model. Lysaker et al. (2010) using a principal component analysis on four purported ToM tests, identified one factor single factor with an Eigen value of 2.26 (i.e., 56.5% of the variance explained) to explain the variance. This value compares favorably with that found in the present study (Eigen value of 2.27) and this despite the fact that the battery included Picture Arrangement (WAIS-III), a test that is not normally used to measure ToM, This concordance contrasts with the results obtained by Mancuso et al. (2011). Using a factor analyses, these authors identified three factors that accounted for 53.6% of the variance using a mixed battery of eight subscales for three measures of social cognition, a test of ToM, a test of attributional style, a test of facial expressions of emotion. The three factors identified relate to the factor structure of social cognition, not to ToM.

It was hypothesized that C&I would be the most sensitive of the ToM tests as this is a dynamic, realistic test requiring on-line encoding, and decoding of complex visual, and auditory, verbal and non-verbal material. Performance on the C&I task was not what was primarily affected in this group of patients. In fact, the clinical group did not appear to be more perturbed by the complexity of the ongoing social interaction in real time than they were by the requirement to read between the lines of a series of written scenarios and discern what the protagonist really meant. The question is why are certain dimensions impaired in a specific pathology and other dimensions less affected? What are the specific symptoms, and pathology that would explain this pattern? In the case of paranoid schizophrenia, one of the symptoms that has been cited is a problem of mentalization (Frith, 1992, 1994; Montag et al., 2011). The picture is less definitive in the present study. In the case of C&I, the percentage of non-interpretative errors made by the clinical group was greater than that of the non-clinical group, while the percentage of incomplete attribution errors was less in the clinical group. As well, within group, the percentage of incorrect attribution errors made by the clinical group was greater than the percentage of incomplete errors. These results confirm at least one result found by Montag et al. (2011). Using a similar format, a short movie task to measure first- and second-order false beliefs, metaphors, faux pas, and sarcasms, these authors also found significantly more literal interpretations of mental states or overly simplistic inferences in the group of PScz patients relative to the non-clinical group but not errors of social inferential reasoning that goes beyond the bounds of the context when controlling for episodic memory and errors on questions requiring reasoning not related to social inferences. There is however, no indication of any difference between the number of reduced ToM and excessive ToM errors in this group.

In the case of the Hinting Task, the percentage of different types of errors in the clinical group did not differ from that in the non-clinical group. In the case of *Strange Stories* and the Faux Pas test, there was also no difference between the two groups and the percentage of non-interpretative errors and incomplete attribution was greater than the percentage of incorrect attributions errors for both groups combined.

Corcoran (2003) presents the argument that the impaired performance can be explained by a problem with inductive reasoning in this population. If this were true one would expect a preponderance of literal interpretations or “I don’t know” answers or that the participants would tend to give more incorrect answers. In fact the clinical group made the same percentage of non-interpretative errors, as incorrect, as incomplete attributions as the non-clinical group. They just made significantly more errors of each type than the non-clinical group.

## Conclusion

These results open the door to the possibility that ToM be subdivided into separable dimensions: first and second order inferences or beliefs, interpretation of intentions, interpretation of affect (Shamay-Tsoory et al., 2005, and on the basis of the social cognitive ToM content: faux pas, interpreting indirect messages, lies, irony etc., and contexts (cf. C&I). The ability to interpret depends on an interaction between the neurologic systems that are affected, the explicit and latent content in the message and the context in which the message is expressed. The social cognitive content to be encoded and interpreted has many dimensions and these dimensions are dissociable within a given pathology or state of mind of the participant. Each social cognitive ToM content inherent in the task adds or subtracts a facet (prosody, facial expressions, gestures, the manifest verbal content) that either facilitates the interpretative process or impairs it. As well, the sensitivity and specificity of the tests may vary between pathologies (Bazin et al., 2009; Eddy et al., 2010; Ouellet et al., 2010). In order to best characterize the deficits it would be advisable to use different tests of ToM that target different content and contexts. Such an approach would help to better inform us of the nature of the deficits and the pathology. Finally, it should be noted that Hinting Task and Strange Stories are probably better clinical instruments than the C&I (or perhaps similar tests), not because they are significantly more sensitive or specific in this population but because they are more economical to use, require less time than C&I, and are more frequently used is research with diverse populations. What is missing are norms stratified for age and education for these two tests, which would increase their clinical and experimental utility.

These results need to be replicated with larger samples and in different social economic groups in order to verify the validity of the findings.

## Conflict of Interest Statement

The authors declare that the research was conducted in the absence of any commercial or financial relationships that could be construed as a potential conflict of interest.
